# Tracing the paths: a systematic review of mediators of complex trauma and complex post-traumatic stress disorder

**DOI:** 10.3389/fpsyt.2024.1331256

**Published:** 2024-03-06

**Authors:** Joseph Harris, Eva Loth, Vaheshta Sethna

**Affiliations:** ^1^ Institute of Psychiatry, Psychology and Neuroscience, King’s College London, London, England, United Kingdom; ^2^ Department of Forensic and Neurodevelopmental Science, School of Academic Psychiatry, King’s College London, London, England, United Kingdom; ^3^ Department of Social, Genetic & Developmental Psychiatry Centre, School of Mental Health & Psychological Sciences, King’s College London, London, England, United Kingdom

**Keywords:** complex trauma, CPTSD, mediator, complex post-traumatic stress disorder (C-PTSD), systematic review, mediation

## Abstract

Complex trauma is associated with complex-posttraumatic stress disorder (CPTSD). While dissociative processes, developmental factors and systemic factors are implicated in the development of CPTSD, there are no existing systematic reviews examining the underlying pathways linking complex trauma and CPTSD. This study aims to systematically review evidence of mediating factors linking complex trauma exposure in childhood (birth to eighteen years of age) and subsequent development of CPTSD (via self-reports and diagnostic assessments). All clinical, at-risk and community-sampled articles on three online databases (PsycINFO, MedLine and Embase) were systematically searched, along with grey literature from ProQuest. Fifteen articles were eligible for inclusion according to pre-determined eligibility criteria and a search strategy. Five categories of mediating processes were identified: 1) dissociative processes; 2) relationship with self; 3) emotional developmental processes; 4) social developmental processes; and 5) systemic and contextual factors. Further research is required to examine the extent to which targeting these mediators may act as mechanisms for change in supporting individuals to heal from complex trauma.

**Systematic review registration:**

https://www.crd.york.ac.uk/PROSPERO/, identifier CRD42022346152.

## Introduction

### Complex post-traumatic stress

Experiences in early life have a lasting impact on psychological development, even if not consciously remembered (1, 2). When these experiences are traumatic – when they overwhelm an individual’s capacity to cope – the consequences are often severe (3). When the trauma is ‘complex’ – when it is repeated and prolonged, as in childhood abuse or domestic violence – it is associated with a complex post-traumatic stress response (4, 5). The ICD-11 conceptualises this as ‘Complex Post-Traumatic Stress Disorder’ (‘CPTSD’) and characterises this response through two domains – a ‘Post-Traumatic Stress Disorder’ domain (‘PTSD’; 1) traumatic re-experiencing; 2) hypersensitivity to potential threat, and; 3) behavioural avoidance of situations which may trigger re-experiencing) and a ‘Disturbances in Self-Organisation’ domain (‘DSO’; 1) emotion dysregulation; 2) a persistent negative self-perception and; 3) interpersonal difficulties; 6).

Prevalence estimates for CPTSD in the general population range from 2.6-7.7% (7, 8; 9) and are higher for at-risk populations such as adults with lived experience of psychological difficulty (12.72%; 10) and refugees (between 2.2 and 50.9%; 11). CPTSD greatly impacts psychosocial functioning, particularly through leading to a fear of relationships, relationship depression, and preoccupations with intimate relationships (12).

Despite this, there is a relative paucity of research investigating the mechanisms through which complex trauma and CPTSD are associated (13). Furthermore, the National Institute for Health and Care Excellence (NICE) do not yet provide specific guidance on evidence-based CPTSD interventions (14). Therefore, there is a need for further research to examine the mechanisms involved in the development of CPTSD to inform clinical understanding and intervention.

### Identifying mechanisms and pathways linking complex trauma and CPTSD

Currently, there are no existing systematic reviews examining the underlying pathways linking complex trauma and CPTSD. Existing systematic reviews and meta-analyses have focused primarily on establishing evidence for the CTPSD construct (15), the prevalence of CPTSD in specific populations (11), and exploring the efficacy of interventions targeting CPTSD (16). While these reviews provide key information, there is a need for further improving understandings of the relationship between complex trauma and CPTSD.

Evidence suggests that factors involving dissociation (4), child development (17), attachment security (18; 19), and wider systemic factors such as family environment (20, 21) may explain the relationship between complex trauma and CPTSD. Due to the nature of these factors and how they theoretically relate to the domains of CPTSD (i.e. interpersonal difficulties), it is possible that some identified mediators may conceptually overlap with CPTSD outcomes. Mediation analyses help identify which factors may influence the effects of an antecedent event (i.e. experiencing complex trauma) towards a particular outcome (i.e. CPTSD; 22). Identifying mediators is therefore one approach to understanding the underlying pathways and mechanisms linking complex trauma and CPTSD, and will provide an important first step in subsequent identification of causal mechanisms in the development of CPTSD (23).

### The current review

This systematic review therefore aims to examine and collate evidence regarding the underlying mechanisms and pathways mediating the relationship between complex trauma and CPTSD. All observational and experimental studies which have examined factors mediating the association between childhood complex trauma and subsequent presentation of CPTSD in childhood, adolescence, or adulthood, will be included.

## Methodology

This review was conducted with the Preferred Items for Systematic Reviews and Meta-Analyses (PRISMA) 2020 guidelines (24) and registered in PROSPERO (CRD42022346152).

### Inclusion and exclusion criteria

The definition of ‘complex trauma’ used was: “Exposure to multiple and/or prolonged traumatic events – often of an invasive, interpersonal nature” (National Child Traumatic Stress Network, 17). Observational and experimental studies were included based on the following inclusion criteria: 1) Clinical, at-risk or community samples in childhood, adolescence, adulthood, older adulthood; 2) Complex trauma experienced during childhood and adolescence (i.e. birth-18 years), assessed with a validated measure – retrospective self-reports and clinical interviews. There were no other timing requirements for trauma exposures; 3) Demonstration of established CPTSD outcomes with validated CPTSD assessments – self-reports and diagnostic assessments; 4) Reporting of mediators linking complex trauma and CPTSD; 5) Inclusion of peer-reviewed articles and grey literature. Exclusion criteria were: 1) Presence of singular or discrete trauma; 2) Articles not written in or translated to English. As previous research has demonstrated that the CPTSD, PTSD and BPD diagnostic constructs describe separate clinical presentations, despite apparent similarities (25, 26), articles solely examining singular-event PTSD and BPD were not included in the inclusion/exclusion criteria or search process.

### Information sources

Three online databases were selected (PsycINFO, MedLine and Embase) based on clinical research emphases. These were searched up to and including 24/06/2023. To reduce article bias associated with solely reviewing published research (27) grey literature was retrieved from ProQuest. A forward and backward search was conducted to ensure all potentially relevant articles were identified. All identified articles were exported to EndNote.

### Search strategy

A search strategy was developed using the PICO framework for systematic reviews to identify studies which examined mediators of the relationship between complex trauma exposure and CPTSD (see **Table 1**; 28). An ‘A’ (‘Analysis’) component was added to the framework to include mediation analyses in the search.

**Table 1:** The search terms used to identify articles which examined mediators of the relationship between complex trauma and CPTSD.

PICO Component	Search Terms
P (Population/Sample)	Complex Post-Traumatic Stress* OR Complex Post-Traumatic Stress Disorder OR CPTSD OR C-PTSD OR Disturbances in Self-Organisation OR DSO OR ICD-11
I (Phenomenon of Interest)	Complex Trauma OR Complex Trauma Exposure OR Child* Maltreatment OR Child* Abuse OR Emotional Abuse OR Emotional Trauma OR Emotion Maltreatment OR Physical Abuse OR Physical Maltreatment OR Sexual Abuse OR Domestic Violence OR Psychological Abuse OR Verbal Abuse OR Neglect OR Victimisation OR Polyvictimisation OR Adverse Childhood Experience* OR ACEs
C (Comparison)	N/A
O (Outcome)	Complex Post-Traumatic Stress* OR Complex Post-Traumatic Stress Disorder OR CPTSD OR C-PTSD OR Traumatic Re-Experiencing OR Traumatic Reexperiencing OR Hyperarousal OR ICD-11 OR Disturbances in Self-Organisation OR DSO OR Dissociation OR Emotion Regulation OR Emotion Dysregulation OR Hypervigilance OR Interpersonal Difficulties OR Relational Di fficulties OR Negative Self-Concept OR Self-Concept
A (Analysis Type)	Mediation OR Mediation Analysis OR Mediat*

### Study selection

The primary author screened the titles and abstracts of all exported articles for eligibility. A random sample of 20% of exported articles were then screened by a separate rater. Inter-rater reliability was very high (Cohen’s κ = 1.00). Following establishment of inter-rater reliability, eligible studies were fully screened by the primary author and another random 20% were screened by the second rater. Again, inter-rater reliability was very high (Cohen’s κ = 1.00).

### Methodological quality

The methodological quality of all included articles was assessed by separate raters via the Effective Public Health Practice Project (EPHPP) Quality Assessment Tool for Quantitative Studies (29). Articles were rated as ‘Overall Strong’ if there were no individual ‘Weak’ ratings, ‘Overall Moderate’ if there was one individual ‘Weak’ rating, and ‘Overall Weak’ if there were two or more individual ‘Weak’ ratings.

### Data extraction and analyses

As shown through the custom data collection form (Appendix A) data was extracted regarding: Article Characteristics; Participants; Design; Exposure Variables; Outcome Variables; and Mediator Variables. Data were then analysed through a narrative synthesis approach (30). This involved: 1) describing results mediator of the association between complex trauma exposure in childhood and CPTSD; 2) constructing mediator categories based on theoretical relationships between identified mediators.

## Results

### Study selection

**Figure 1:**
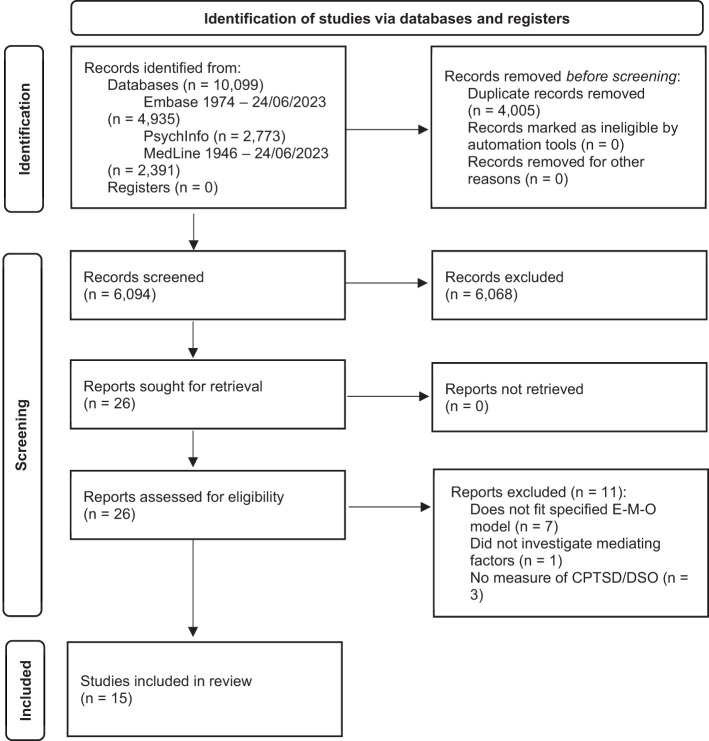
PRISMA (2020) Flow Diagram for Systematic Review Search Process.

The final review consisted of fifteen articles. The results at each stage of the search and screening process are represented in **Figure 1**.

### Quality assessment

Through the EPHPP Guidelines (29), the majority of studies were assessed as having overall “Moderately Strong” methodologies (*k* = 9). The remaining studies were assessed as having overall “Weak” methodologies (*k* = 6). Detailed ratings from the quality assessment are provided in **Table 2**.

**Table 2:** Results of the quality assessment of studies included in the review using the EPHPP Quality Assessment Tool.

	Global Rating	Selection Bias	Study Design	Confounders	Blinding	Data Collection	Withdrawal
31	Weak	Strong	Weak	Weak	Moderate	Strong	Strong
32	Moderate	Moderate	Weak	Strong	Moderate	Strong	Strong
33	Moderate	Moderate	Weak	Moderate	Moderate	Strong	Moderate
34	Moderate	Strong	Weak	Strong	Moderate	Strong	N/A
35	Weak	Strong	Weak	Weak	Moderate	Strong	Strong
36	Weak	Strong	Weak	Weak	Moderate	Strong	Strong
37	Moderate	Strong	Weak	Strong	Moderate	Strong	N/A
38	Weak	Strong	Weak	Weak	Moderate	Strong	Strong
39	Strong	Moderate	Moderate	Moderate	Moderate	Strong	Moderate
40	Weak	Strong	Weak	Strong	Moderate	Strong	Weak
41	Moderate	Moderate	Weak	Strong	Moderate	Strong	N/A
42	Moderate	Strong	Weak	Strong	Moderate	Strong	Moderate
13	Weak	Strong	Weak	Weak	Moderate	Strong	Moderate
43	Strong	Strong	Moderate	Strong	Moderate	Strong	Moderate
44	Moderate	Strong	Weak	Moderate	Moderate	Strong	Moderate

Overall methodological quality was determined in line with the EPHPP guidelines, as follows: Strong = No ‘Weak’ ratings; Moderate = One ‘Weak’ rating; Weak = Two or more ‘Weak’ ratings. N/A = 'Not Applicable' due to the nature of the study design/methods used.

### Sample characteristics

The majority of articles were European, with one article published in China and one article published in the USA. A mixture of clinical (*k* = 7), at-risk (*k* =5) and community samples (*k* = 3) were utilised. At-risk samples experienced social adversities such as being looked after in foster care facilities, experiencing homelessness, experiencing enforced occupation measures, and previous experience of complex trauma. Participants varied greatly in age, ranging from adolescence to older adulthood at time of participation (mean age = 40.27 years, SD = 10.47, range = 14-77). Participants had varying levels of educational attainment (i.e. secondary school, university-educated and post-graduate educated) and a range of marital statuses (i.e. single, partnered, married). Only three articles reported information on participants’ racial backgrounds and five articles reported information on participants’ geographical backgrounds. In articles where these variables were reported, participants came from a variety of racial (i.e. white, Latino, Asian, black, mixed) and geographical backgrounds (i.e. Austrian, UK, Western Europe, African Caribbean, African). No articles reported information on participants’ sexualities.

The majority of articles described studies utilising a cross-sectional design (*k*= 14), with one study utilising a case-control study design. There was considerable heterogeneity in the types of statistical analyses undertaken (i.e. simple mediation analyses, multiple mediation analyses, path analyses, multigroup path analyses, network analysis), with further variation in the reporting of outcomes. Despite this, several mediators of the relationship between complex trauma and CPTSD were identified consistently across articles, allowing for the meaningful categorising of mediators. All articles were published from 2013 onwards.

### Complex trauma *(exposure)* and complex post-traumatic stress disorder *(outcome)*

Complex trauma was mainly assessed retrospectively through self-reports, the most common being the Childhood Trauma Questionnaire (CTQ; 18). All articles identified childhood complex trauma which occurred during childhood, but only one study distinguished between specific exposure timepoints (i.e. childhood and adolescence; 34). The primary identified type of complex trauma was childhood abuse (physical, emotional and sexual) and neglect (physical and emotional). All articles reported an association between complex trauma and CPTSD. CPTSD was mainly assessed through self-report questionnaires, most commonly with the International Trauma Questionnaire (45). The majority of articles (*k*= 9) examined the PTSD and DSO domains of CPTSD as distinct variables, whereas the remaining articles (*k*= 6) examined CPTSD as a composite variable comprising both domains.

### Mediators of complex trauma and CPTSD

Through a narrative synthesis approach, twenty-four mediators of the relationship between complex trauma and CPTSD were identified and described. These were categorised as: 1) ‘Dissociative Processes’, 2) ‘Relationship with Self, 3) ‘Emotional Development’, 4) ‘Social Development’, and 5) ‘Systemic and Contextual Factors’. Each category contained a variety of risk and protective factors. **Table 3** details each mediator group and mediator.

**Table 3:** Descriptions of mediator categories and individual mediators of complex trauma and complex post-traumatic stress disorder (CPTSD).

Mediating Variables	Definitions	Identifying Articles
**Dissociation**	Dis-integration of aspects of experience (e.g. emotions, somatosensory information, beliefs etc.) from consciousness (46).	
Dissociation	(Definition as above).	34; 33.
Psychoform Dissociation	Dis-integration specific to psychological phenomena (e.g. cognition; 43).	13, 43.
Somatoform Dissociation	Dis-integration specific to somatosensory phenomena (e.g. physiological changes in the body, functional symptoms; 43).	43
**Relationship with Self**	An individual’s ongoing relationship with their self.	
Negative Self Relationship (Alterations in Self- Perception; Self- Judgement; Early Maladaptive Schema; Disconnection Schema; Impaired Autonomy Schema)	A negatively valenced relationship with oneself, characterised by self-criticism and judgement.	31, 32, 44.
Positive Self Relationship (Self- Compassion; Self-Kindness)	A positively valenced relationship with oneself, characterised by compassion and acceptance.	32; 40
Sense of Coherence	A belief that life is meaningful and manageable with the skills an individual has available to them (42).	42).
**Emotional Development**	Development of capacities relating to emotional experience, such as emotion regulation and understanding of emotions.	
Emotion Regulation (Emotion Regulation; Adaptive Emotion Regulation; Under- Regulation of Affect; Impulsivity; Strategies for Emotion Regulation)	Overall capacity to adaptively regulate trajectories of emotional experiencing (47).	33, 36, 43).
Emotional Clarity	Whether an individual has clarity (understanding) of their emotions (48).	36
**Social Development**	Development of social capacities, including attachment relationships and use of them, interpersonal skills, and ability to mentalise the minds of others.	
Attachment Insecurity (Attachment Anxiety; Attachment Avoidance)	The development of an insecure attachment style, linking to beliefs that others cannot be trusted or depended on and behaviours reflecting this anxiety (e.g. anxious pursuit or avoidant behaviour; 49).	39, 41
Personality Functioning	A model of personality examining the intersection of self-perception, interpersonal contact and internal models of relationships.	35
Relational Fears (Fear of Abandonment; Fear of Closeness)	Relational fears arising from negative expectancies of interpersonal relationships, resulting from early attachment injuries (50).	43
**Systemic and Contextual Factors**	Factors pertaining to the systems a traumatised individual is embedded within (e.g. family, culture etc.) and the context of their life.	
Social Acknowledgement (Social Acknowledgement; Social Disapproval)	Whether there is acknowledgement or disapproval by the family of the individual’s experience of trauma.	37–39
Social Support	Perceived levels of emotional and practical support for the traumatised person (39).	39
Factors Impacting Disclosure of Trauma (Avoidance of Trauma Disclosure; Disclosure of Trauma; Dysfunctional Disclosure)	It is not safe – psychologically or physically – in all environments to disclose experiences of trauma.	37–39

The majority of articles described controlled for confounding variables such as gender, age and social desirability. Inferential statistics for each mediation effect are shown in **Tables 4**, **5** and **6**. A range of small, medium and large effect sizes were identified.

**Table 4:** Extracted data on study characteristics and results from articles with an EHPP Global Rating of **‘Strong’**.

Article Characteristics	Participants	Design	Exposure Variable(s)	Outcome Variable(s)	Mediator Variable(s)	
First Author, Year, Country of Publication	**Population** **Sample** N (% Female) Mean Age (SD) Key Demographics	**Study Design** **Analysis**	**Complex Trauma Variables:** Trauma Type **Measure Type:** Measure	**CPTSD Outcomes**: % of Sample with CPTSD **Measure Type:** Measure	**Mediator Category:** Identified Mediator(s) **Measure Type:** Measure **Factor Type:** Risk/Protective Factor	**Results of Mediation Analyses** **Effect Size**
39, Switzerland.	**Population:** At-risk sample recruited from a national programme for individuals affected by compulsory social measures in Switzerland. **Sample:** 251 (46.45% female). 70.68 years (SD = 11). 23.9% never married; 47.01% average monthly income >4,670 (CHF).	**Study Design:** Case control. **Analysis:** Multigroup Path Analysis.	**Complex Trauma Variables:** Emotional Abuse, Physical Abuse, Sexual Abuse, Emotional Neglect, Physical Neglect. ***Self-Report Questionnaire:*** CTQ (51); TEC (52).	**CPTSD Outcomes:** 5% CPTSD. ***Self-Report Questionnaire:*** ITQ (45).	**Relationship to Self:** Self-Efficacy. ***Self-Report Questionnaire:*** General Self-Efficacy (GSE; 53). **Social Development:** Attachment Insecurity (Avoidance and Anxiety). ***Self-Report Questionnaire:*** Experiences in Close Relationships-Revised Questionnaire (ECR-R; 54). **Systemic and Contextual Factors:** Social Acknowledgement; Social Support; Disclosure of Trauma. ***Self-Report Questionnaire:*** Social Acknowledgement Questionnaire (SAQ; 55); German Social Support Questionnaire-Short Version (F-SozU; 56); Disclosure of Trauma Questionnaire (DTQ; & Maercker, 2006). **Factor Type:** Self-Efficacy – Protective Factor Attachment Insecurity – Risk Factor Social Acknowledgement – Protective Factor Social Support – Protective Factor Disclosure of Trauma – Protective Factor	**Results of Mediation Analysis:** A non-exhaustive list of key multiple mediation paths are described below. **First Example Mediation Path:** Emotional Abuse -> Attachment Anxiety (**β = -0.20**, 95% CI = -0.39-0.02, *p* = <.001) -> Disclosure of Trauma (**β = 0.27**, 95% CI = 0.05-0.49, *p* = .015) -> Core PTSD Symptoms (**β = 0.38**, 95% CI = 0.22 – 0.53, *p* = <.001). **Second Example Mediation Path:** Emotional Neglect -> Attachment Anxiety (**β = -0.30**, 95% CI = 0.15 – 0.45, *p* = <.001) -> Disclosure of Trauma (**β = -0.42**, 95% CI = -0.58 - -0.26, *p* = <.001) -> DSO (**β = 0.42**, 95% CI = 0.30 – 0.54, *p* = <.001). **Third Example Mediation Path:** Physical Neglect -> Attachment Anxiety (**β = 0.08**, 95% CI = -0.48 - -0.8, *p* = .007) -> Social Support (**β = -0.54**, 95% CI = -0.70 - -0.37, *p* = <.001) -> DSO (**β = -0.31**, 95% CI = -0.51 - -0.11, *p* = .001).
43, Netherlands.	**Population:** Clinical sample recruited from psychiatric inpatient hospital. **Sample:** 449 (70.05% female). 34.05 years (SD = 10.05). 24.83% no primary partner; 17.48% higher education; 12.91% diagnoses of borderline personality disorder.	**Study Design:** Case-control. **Analysis:** Mediation analysis with path analysis.	**Complex Trauma Variables:** Childhood trauma. ***Self-Report Questionnaire:*** Traumatic Experiences Checklist (52).	**CPTSD Outcomes:** 28% CPTSD. ***Self-Report Questionnaire:*** SIDES-rev (57).	**Dissociation:** Negative Psychoform Dissociation; Positive Somatoform Dissociation. ***Self-Report Questionnaire:*** Dissociative Experiences Scale (DES; 58); Somatoform Dissociation Questionnaire (SDQ-20; 52). **Emotional Development:** Under-Regulation of Affect. ***Self-Report Questionnaire:*** Bermond Vorst Alexithymia Questionnaire (BVAQ; 59); Affect instability scale from Personality Disorder Severity Index (BDSI; 60). **Social Development:** Fear of Abandonment; Fear of Closeness. ***Self-Report Questionnaire:*** Relationship Style Questionnaire (RSQ; 61). **Factor Type:** Negative Psychoform Dissociation - Risk Factor Positive Somatoform Dissociation – Risk Factor Under-Regulation of Affect – Risk Factor Fear of Abandonment – Risk Factor Fear of Closeness – Risk Factor	**Results of Mediation Analysis:** Negative psychoform dissociation (*b* = .15, BS 95% CI = .06 -.24, *SE b* = .05, **β = .05,** *p* = .002) mediated the path between CTE and CPTSD. Positive somatoform dissociation did not reach significance but was considered a trend (*b* = .04, BS 95% CI = -.01 -.09, *SE b* = .02, **β = .01**, *p* = .08). Under-regulation of affect mediated paths from CTE to CPTSD (***b* = .24**, bootstrapped 95% CI = .14 -.34, SE *b* = .05, **β = .08**, *p* <.001). Fear of abandonment mediated paths from CTE to CPTSD (*b* = .10, bootstrapped 95% CI = 03 -.17, SE *b* = .04, **β = .03**, *p* <.007). Fear of closeness mediated paths from CTE to CPTSD (*b* = .11, bootstrapped 95% CI = .03 -.20, SE *b* = .05, **β = .04**, *p* <.001).

Numbers in bold indicate an effect size.

**Table 5:** Extracted data on study characteristics and results from articles with an EHPP Global Rating of **‘Moderate’**.

Article Characteristics	Participants	Design	Exposure Variable(s)	Outcome Variable(s)	Mediator Variable(s)	
First Author, Year, Country of Publication	**Population** **Sample** N (% Female) Mean Age (SD) Key Demographics	**Study Design** **Analysis**	**Complex Trauma Variables:** Trauma Type **Measure Type:** Measure	**CPTSD Outcomes**: % of Sample with CPTSD **Measure Type:** Measure	**Mediator Category:** Identified Mediator(s) **Measure Type:** Measure **Factor Type:** Risk/Protective Factor	**Results of Mediation Analyses** **Effect Size**
32, China.	**Population:** Clinical sample of university students recruited online. **Sample:** 1,361 (65.83% female). 20.73 years (SD = 1.88). 32.7% no partner; 67.3% had siblings; 42% from rural areas.	**Study Design:** Cross-sectional. **Analysis:** Structural Equation Modelling.	**Complex Trauma Variables:** Adverse Childhood Experiences ***Self-Report Questionnaire:*** Revised Adverse Childhood Experiences Scale (62).	**CPTSD Outcomes:** % Not Reported. ***Self-Report Questionnaire:*** ITQ (45).	**Relationship to Self:** Self-Judgement (SJ); Self-Kindness (SK). ***Self-Report Questionnaire:*** Self-Compassion Scale-Short Form (63). **Factor Type:** SJ – Risk Factor SK – Protective Factor	**Results of Mediation Analysis:** SJ significantly mediated the relationship between ACEs and PTSD/DSO (**β = .03**, 95% CI = .02 -.06, *p* = <.001). SK significantly mediated the relationship between ACEs and PTSD/DSO (**β = .06**, 95% CI = .04 -.09, *p* = <.001).
33, Austria.	**Population:** At-risk sample recruited from foster care facilities. **Sample:** 122 (42.6% female). 14.47 years (SD = 2.24) 88.5% Austrian-born; 37.8% attending secondary school; 34.2% special needs.	**Study Design:** Cross-sectional. **Analysis:** Network analysis.	**Complex Trauma Variables:** Emotional Abuse; Physical Abuse; Sexual Abuse; Cumulative Abuse. ***Self-Report Questionnaire:*** Childhood Trauma Questionnaire (51).	**CPTSD Outcomes:** 10.7% CPTSD ***Self-Report Questionnaires:*** International Trauma Questionnaire (45).	**Emotional Development:** Adaptive Emotion Regulation ***Self-Report Questionnaires:*** Questionnaire to Assess Children and Adolescent’s Emotion Regulation (64). **Factor Type:** Protective Factor	**Results of Mediation Analysis:** The shortest paths from cumulative childhood trauma (CCT) to CPTSD were mediated by dissociation (CCT to PTSD) and adaptive emotion regulation (CCT to DSO). The 95% CIs around the edge-weights did not include zero.
34, Ireland	**Population:** Community sample. **Sample:** 1,020 (51% female) 43.10 years (SD = 15.12) Nationally representative sample of Irish adults; 69.5% in committed relationships; 59.4% had children; 36.9% university educated; 45.8% in full-time employment.	**Study Design:** Cross-sectional. **Analysis:** Regression modelling.	**Complex Trauma Variables:** Childhood Trauma; Adolescent Trauma; Adulthood Trauma, Total Lifetime Trauma. ***Self-Report Questionnaire:*** International Trauma Exposure Measure (ITEM; 8).	**CPTSD Outcomes:** 8.1% CPTSD. ***Self-Report Questionnaire:*** International Trauma Questionnaire (ITQ; 45).	**Dissociation:** Dissociation. ***Self-Report Questionnaire:*** Dissociation subscale of Trauma Symptom Inventory (Briere, 1996; Self-Report). **Factor Type:** Risk Factor	**Results of Mediation Analysis:** Dissociation mediated all associations between complex trauma (at all timepoints) and PTSD/DSO symptoms. The strongest indirect effect through Childhood Trauma exposure for PTSD (**β = .127**, 95% BS CI = .087 -.163, *p* <.001) and DSO (**β = .142**, 95% BS CI = .100 -.184, *p* <.001).
37, Switzerland.	**Population:** At-risk sample recruited through larger research project on long-term effects of complex trauma. **Sample:** 116 (40.5% female). 77.0 years (SD = 7.1). 40% married, 46.6% living alone, 45.7% living with partner/friend, 7.8% in senior residence home.	**Study Design:** Cross-sectional. **Analysis:** Multiple regression modelling.	**Complex Trauma Variables:** Emotional Abuse; Physical Abuse; Sexual Abuse; Emotional Neglect; Physical Neglect. ***Self-Report Questionnaire:*** CTQ (51).	**CPTSD Outcomes:** % Not Reported. ***Self-Report Questionnaire:*** Trauma Symptom Inventory (65).	**Systemic and Contextual Factors:** Social Acknowledgement (SA); Dysfunctional Disclosure (DD). ***Self-Report Questionnaire:*** SAQ (55); DTQ (66). **Factor Type:** SA – Protective Factor DD – Risk Factor	**Results of Mediation Analysis:** SA significantly mediated CTE and anxious arousal (***R^2^* = .08**, β = .28, *p* = <.05), depression (***R^2^* = .11**, β = -.25, *p* = <.05), anger/irritability (***R^2^* = .12**, β = -.27, *p* = <.01), intrusive experiences (***R^2^* = .08**, β = .27, *p* = <.01), defensive avoidance (***R^2^* = .018**, β = .22/-.25, *p* = <.05/<.01), dissociation (***R^2^* = .11**, β = .22/-.25, *p* = <.05/<.01) and impaired self-reference (***R^2^* = .10**, β = -.28, *p* = <.01). DD significantly mediated CTE and anxious arousal (***R^2^* = .17**, β = .25/.32, *p* = <.01/<.001), depression (***R^2^* = .31**, β = .51, *p* = <.001), anger/irritability (***R^2^* = .08**, β = .21, *p* = <.05), intrusive experiences (***R^2^* = .23**, β = .22/.42, *p* = <.05/<.001), defensive avoidance (***R^2^* = .28**, β = .19/.43, *p* = <.05/<.001), dissociation (***R^2^* = .19**, β = .24/.35, *p* = <.05/<.001) and impaired self-reference (***R^2^* = .12**, β = .33, *p* = <.01).
41, USA.	**Population:** Community sample of university students. **Sample:** 169 (74% female). 19.27 years (SD = 2.40). 39% Latino, 25% Asian, 18% mixed/other, 10% white, 9% black; 96% single.	**Study Design:** Cross-sectional. **Analysis:** Path analysis.	**Complex Trauma Variables:** Interpersonal Trauma; Non-Interpersonal Trauma ***Self-Report Questionnaire:*** Modified version of LEC for DSM-5 (LEC-5; 67).	**CPTSD Outcomes:** 13% CPTSD ***Self-Report Questionnaire:*** ITQ (45).	**Social Development:** Attachment Anxiety. ***Self-Report Questionnaire:*** Experiences in Close Relationships-Revised (ECR-R; 54). **Factor Type:** Attachment Anxiety – Risk Factor	**Results of Mediation Analysis:** Attachment anxiety significantly mediated paths from interpersonal trauma to PTSD (***R^2^* = .40**, *p* <.0001) and DSO (***R^2^* = .35**, *p* = <.0001). Attachment avoidance did not significantly mediate trauma and PTSD/DSO. Non-interpersonal trauma did not have any indirect effects on PTSD/DSO.
42, Austria.	**Population:** At-risk sample recruited through foster care services **Sample:** 140 (41.5% female). 14.24 years (SD = 2.27). 87.2% Austrian-born; 2.6% German; 10.2% from Romania, Russia, Switzerland, Czech Republic, Thailand and USA). 30% attended secondary school, 38.8% attended a special needs school.	**Study Design:** Cross-sectional. **Analysis:** Regression modelling.	**Complex Trauma Variables:** Emotional Abuse; Physical Abuse; Sexual Abuse; Emotional Neglect; Physical Neglect. ***Self-Report Questionnaire:*** CTQ (51).	**CPTSD Outcomes:** % Not Reported. ***Self-Report Questionnaire:*** ITQ (45).	**Relationship to Self:** Sense of Coherence. ***Self-Report Questionnaire:*** Questionnaire for Resources in Children and Adolescents (Lohaus et al., 2017). **Factor Type:** Sense of Coherence – Protective Factor	**Results of Mediation Analysis:** Sense of coherence significantly mediated the effect of CTE on DSO (*b* = 1.28, 95% CI = .045 -.211, *p* = <.05) but not PTSD. ***R^2^* = .04**.
44 England.	**Population:** Clinical sample recruited through Older Adult Community Mental Health services. **Sample:** 42 (73.8% female). 71.5 years (SD = 4.6). 100% White; 90.5% retired; 40.5% married, 14.3% widowed, 21.4% single; 40.5% income lower than 14,999.	**Study Design:** Cross-sectional. **Analysis:** Multiple regression modelling.	**Complex Trauma Variables:** Emotional Abuse; Physical Abuse; Sexual Abuse; Emotional Neglect; Physical Neglect. ***Self-Report Questionnaire:*** CTQ (51).	**CPTSD Outcomes:** 31% CPTSD. ***Self-Report Questionnaire:*** ITQ (45).	**Relationship to Self:** Early Maladaptive Schema; Disconnection Schema; Autonomy Schema. ***Self-Report Questionnaire:*** Young Schema Questionnaire-Short Form 3^rd^ Edition (68). **Factor Type:** Early Maladaptive Schema - Risk Factor Disconnection Schema - Risk Factor Autonomy Schema – Protective Factor	**Results of Mediation Analysis:** EMS significantly mediated the relationship between CTE and CPTSD symptoms (**β = .39**, *SE* = .15, 95% CI = .08 -.67). CTE significantly predicted EMS (*B* = .6, *p* = .001), which in turn significantly predicted CPTSD symptom severity (*B* = .5, *p* = .001). The mediation model accounted for 65% of the variance in CPTSD symptoms (***R* ^2^ = .65**; *F* (3,38) = 24.02, *p* <.001). The indirect effect of EMS had a large effect size (***k* ^2^ = 0.48**). Disconnection schema explained 67% of the variance in CPTSD scores (*F* (3, 38) = 26.16, *p* <.001) with a significant indirect effect (**β = .37**, *SE* = .14, 95% CI = .09 -.63, ***k* ^2^ = 0.46**). Impaired Autonomy schema explained 60% of the variance in CPTSD scores (*F* (3, 38) = 19.23, *p* <.001) with a significant indirect effect (**β = .32**, *SE = .*11, 95% CI = .07 -.52, ***k* ^2^ = 0.38**).

Numbers in bold indicate an effect size.

**Table 6:** Extracted data on study characteristics and results from articles with an EHPP Global Rating of **‘Weak’**.

Article Characteristics	Participants	Design	Exposure Variable(s)	Outcome Variable(s)	Mediator Variable(s)	
First Author, Year, Country of Publication	**Population** **Sample** N (% Female) Mean Age (SD) Key Demographics	**Study Design** **Analysis**	**Complex Trauma Variables:** Trauma Type **Measure Type:** Measure	**CPTSD Outcomes**: % of Sample with CPTSD **Measure Type:** Measure	**Mediator Category:** Identified Mediator(s) **Measure Type:** Measure **Factor Type:** Risk/Protective Factor	**Results of Mediation Analyses** **Effect Size**
31, Ireland	**Population:** Clinical sample of clients attending therapy for complex trauma. **Sample** 44 (20.45% female) 43 years (SD = NR) **Key Demographics** NR	**Study Design:** Cross-sectional. **Analysis:** Regression modelling.	**Complex Trauma Variables:** Emotional Abuse; Physical Abuse; Sexual Abuse; Emotional Neglect; Physical Neglect. ***Self-Report Questionnaires:*** Childhood Trauma Questionnaire (51; Self-Report).	**CPTSD Outcomes:** 77% CPTSD ***Self-Report Questionnaires:*** Post-Traumatic Diagnostic Scale (69); Structured Interview for Disorders of Extreme Stress (SIDES; 70); Self-Harm Behaviour Questionnaire (71)	**Relationship to Self:** Alterations in Self-Perception. ***Self-Report Questionnaires:*** SIDES (70). **Factor Type:** Risk Factor	**Results of Mediation Analysis:** Alterations in self-perception significantly mediated the relationship between physical neglect and self-harm (omnibus *χ* ^2^ = 17.53, *df* = 3, *p* = .001) and accounted for 46% of the variance in the model. ***R^2^* = .46**
35, Germany.	**Population:** Community sample from demography research study. **Sample:** 2,004 (52.5% female). 51.3 years (SD = 18.1). 30.2% <10 years education; 45.5% married, 28.6% single; 41.4% full-time employed, 8% unemployed.	**Study Design:** Cross-Sectional. **Analysis:** Structural equation modelling.	**Complex Trauma Variables:** Adverse Childhood Experiences. ***Self-Report Questionnaire:*** Adverse Childhood Experiences Questionnaire-German Version (72).	**CPTSD Outcomes:** 4.1% CPTSD. ***Self-Report Questionnaire:*** International Trauma Questionnaire (45).	**Social Development:** Personality Functioning ***Self-Report Questionnaire:*** Operationalised Psychodynamic Diagnosis Structure Questionnaire-Short Form (OPD-SQ; 73). **Factor Type:** Risk Factor	**Results of Mediation Analysis:** Including Personality Functioning as a mediator (***β* = .58**) in the relationship between ACEs and CPTSD increased variance explained from 20% to 47%.
36, Scotland.	**Population:** Clinical sample recruited through National Health Service trauma centre. **Sample:** 193 (65.1% female). 40.7 years (SD = 12.4). 88.7% UK-born; 20.2% full-time employment; 30.2% university-educated; 48.2% no partner; 41% living with family.	**Study Design:** Cross-Sectional. **Analysis:** Path Analysis	**Complex Trauma Variables:** Emotional Abuse; Physical Abuse; Sexual Abuse; Emotional Neglect; Physical Neglect. ***Self-Report Questionnaire:*** Childhood Trauma Questionnaire (74); Life Events Checklist (67).	**CPTSD Outcomes:** 53% CPTSD. ***Self-Report Questionnaire:*** International Trauma Questionnaire (45).	**Emotional Development:** Emotion Regulation (ER); Impulsivity; Strategies for ER; Emotional Clarity. ***Self-Report Questionnaire:*** Difficulties in Emotion Regulation Scale (DERS; 48) **Factor Type:** ER – Protective Factor Impulsivity – Risk Factor Strategies for ER – Protective Factor Emotional Clarity – Protective Factor	**Results of Mediation Analysis:** Total ER mediated paths from child abuse to PTSD (***b* = 0.26**, *SE* = 0.06, 95% CI = 0.15 – 0.39) and DSO (***b* = 0.21**, *SE* = 0.09, 95% CI = 0.03 – 0.39). Total ER mediated path from child neglect to PTSD (***b* = 0.24**, *SE* = 0.08, 95% CI = 0.09 – 0.42) but not to DSO. Impulsivity mediated paths from child abuse to PTSD (***b* = 0.03**, *SE* = 0.02, 95% CI = <0.01 – 0.08, *p* = .045) and DSO (***b* = 0.03**, *SE* = 0.02, 95% CI = <0.01 – 0.03, *p* = .002). Emotional Clarity mediated paths from child neglect to DSO (***b* = 0.02**, *SE* = 0.02, 95% CI = <0.01 – 0.07, *p* = .040). Strategies for ER mediated paths from child abuse to DSO (***b* = 0.10**, *SE* = 0.04, 95% CI = 0.04 – 0.19, *p* = <0.001).
38, Lithuania.	**Population:** Clinical sample recruited from larger research project on ICD-11 stress-related disorders. **Sample:** 280 (77.5% female). 39.48 years (SD = 13.35). 79.3% urban; 63.9% employed; 37.9% university educated.	**Study Design:** Cross-sectional. **Analysis:** Structural equation modelling.	**Complex Trauma Variables:** Emotional Abuse, Physical Abuse, Sexual Abuse. ***Self-Report Questionnaire*:** LEC (67).	**CPTSD Outcomes:** 10% CPTSD. ***Self-Report Questionnaire*:** ITQ (45).	**Systemic and Contextual Factors:** Social Disapproval; Avoidance of Trauma Disclosure. ***Self-Report Questionnaire:*** Social Acknowledgement Questionnaire (SAQ; 55); Disclosure of Trauma Questionnaire (DTQ; 66). **Factor Type:** SD – Risk Factor ATD – Risk Factor	**Results of Mediation Analysis:** Social disapproval (***R^2^ =* 0.02**) and avoidance of trauma disclosure (***R^2^ =* 0.3**) significantly mediated CTE and CPTSD. The model did not significantly differ from the data (*χ* ^2^ (14) = 19.91, *P* = 0.133, CFI/TLI = 0.972/0.944, RMSEA 90% C.I. 0.039 (0.000-0.076), SRMR = 0.051).
40, Ireland.	**Population:** At-risk sample recruited through homelessness hostels and day-services. **Sample:** 56 (21% female). 37.11 years (SD = 9). 85.71% Irish; 1.79% Filipino, Somalian and Romanian; 7.14% rough sleeping; 66.07% hostel accommodation; 35.7% in a relationship.	**Study Design:** Cross-sectional. **Analysis:** Regression modelling.	**Complex Trauma Variables:** Interpersonal Trauma (e.g. Abuse); Non-Interpersonal Trauma. **Adulthood Trauma:** Interpersonal Trauma; Non-Interpersonal Trauma. ***Self-Report Questionnaire:*** ITEM (8).	**CPTSD Outcomes:** 34% CPTSD. ***Self-Report Questionnaire:*** ITQ (45).	**Relationship to Self:** Self-Compassion. ***Self-Report Questionnaire:*** Self-Compassion Scale Short-Form (63). **Factor Type:** Self-Compassion – Protective Factor	**Results of Mediation Analysis:** Self-compassion significantly mediated the total effect of CTE on CPTSD severity (*B* = 3.40, *SE* = 0.79, *p* = .0001). ***R* ^2^ = 0.34**.
13, Netherlands	**Population:** Clinical sample recruited from psychiatric inpatient hospital. **Sample:** 472 (69.27% female). 34.7 years (SD = 10.1). 37.9% no partner; 50% lived with partner; 34.5% high-level secondary education.	**Study Design:** Cross-sectional. **Analysis:** Mediation Analysis; Path Analysis.	**Complex Trauma Variables:** Emotional Abuse; Physical Abuse; Sexual Abuse; Emotional Neglect; Physical Neglect. ***Self-Report Questionnaire:*** Traumatic Experiences Checklist (TEC; 52).	**CPTSD Outcomes:** NR (63% with Complex Childhood Trauma). ***Self-Report Questionnaire:*** Structured Interview for Disorders of Extreme Stress Not Otherwise Specified (SIDES-rev; 57).	**Dissociation:** Psychoform Dissociation. ***Self-Report Questionnaire:*** Dissociative Experiences Scale (DES; 58); Somatoform Dissociation Questionnaire (SDQ-20; 52). **Factor Type:** Psychoform Dissociation – Risk Factor	**Results of Mediation Analysis:** Psychoform dissociation partially mediated the association between CCT and CPTSD symptom severity (***b* = 3.70**, 95% CI = 1.99 – 5.71, *p* = <.05).

Numbers in bold indicate an effect size.

### Dissociation

Four articles examined dissociation, defined either as a single process (i.e. ‘dissociation’) or as two sub-processes (‘psychoform’ and ‘somatoform’ dissociation). All four articles identified statistically significant mediation effects of dissociation in a variety of geographical samples, indicating cross-cultural effects: a nationally representative community sample in Ireland, an at-risk sample of adolescents in foster care in Austria, and two clinical samples from psychiatric inpatient services in the Netherlands. It was found that the strongest mediation effect occurred when exposure to complex trauma was during childhood (as opposed to during adolescence), that dissociation mediated paths specifically from exposure to the PTSD symptom cluster of CPTSD (but not to the DSO symptom cluster), and specifically that the psychoform subtype of dissociation mediates complex trauma and CPTSD association. This effect was identified as being independent of the relationship between complex trauma and BPD.

### Relationship with self

Five articles examined processes linked to one’s relationship to self: self-judgement, self-kindness, self-compassion, sense of coherence, early maladaptive schema and alterations in self-perception. Self-compassion was identified as a statistically significant mediator of complex trauma (e.g. abuse) and CPTSD both in a sample of adults in Ireland experiencing homelessness and a community sample of university students in China, demonstrating a cross-cultural effect. Of these, one article demonstrated further specific mediation effects of self-compassion on the associations between complex trauma and both the PTSD and DSO domains of CPTSD. In this same sample of university students, self-judgement additionally mediated the associations between complex trauma and the PTSD and DSO domains. In a clinical sample of older adults accessing community mental health services in England, early maladaptive schemas were further found to mediate the relationship between complex trauma and CPTSD with medium-to-large effect sizes. Other articles indicated a mediation effect of self-related factors more specifically between complex trauma and the DSO domain. In an at-risk sample of adolescents in foster care in Austria, sense of coherence mediated the relationship between complex trauma and the DSO domain but not the PTSD domain. Similarly, in a clinical sample of adults in Ireland attending therapy for complex trauma, alterations in self-perception mediated the relationship between complex trauma exposure and a specific form of DSO (i.e. self-harm).

### Emotional development

Three articles examined the mediating role of emotional development. Firstly, in a clinical sample of psychiatric inpatients, under-regulation of affect was identified as a mediator of complex trauma exposure and CPTSD. This mediation effect was independent of the association between complex trauma and BPD. Similarly, in an at-risk sample of adolescents in foster care, adaptive emotion regulation was found to be a mediator of the association between exposure and DSO. Lastly, using an adult clinical sample in Scotland, another article identified more specific emotional developmental processes which mediated specific forms of complex trauma and the PTSD and DSO domains: total emotion regulation mediated relationships between child abuse and PTSD/DSO, and mediated the link between child neglect and PTSD; impulsivity mediated the relationship between child abuse, PTSD and DSO; emotional clarity mediated the relationship between child neglect and DSO, and strategies for emotion regulation mediated the relationship between child abuse and DSO.

### Social development

Four articles examined the mediating role of social development: personality functioning, attachment anxiety, attachment avoidance, fear of abandonment, and fear of closeness. In a nationally representative community sample in Germany, a primarily interpersonal model of personality functioning was found to mediate complex childhood trauma and CPTSD in adulthood at a large effect size. In both a community sample of university students and an at-risk sample of adults, attachment anxiety significantly mediated the relationship between interpersonal trauma and DSO, and was involved in multiple mediation paths from emotional abuse, emotional neglect and physical neglect to PTSD and DSO. Furthermore, in a clinical sample of inpatients in a psychiatric hospital in the Netherlands, the relationship between complex trauma exposure and CPTSD was significantly mediated by fear of abandonment and fear of closeness. These mediation effects were identified as independent of the association between complex trauma and BPD.

### Systemic and contextual factors

Three articles examined systemic and contextual factors: social disapproval, avoidance of trauma disclosure, social acknowledgement, social support, disclosure of trauma and dysfunctional disclosure. Social disapproval of close family or friends and avoidance of trauma disclosure were found to significantly mediate the association between complex trauma exposure and CPTSD in both a clinical sample and an at-risk sample of adults. In this same at-risk sample, lack of social support was found to mediate complex trauma in childhood and DSO in adulthood. In another at-risk sample of adults, social acknowledgement and dysfunctional disclosure of trauma significantly mediated the following aspects of the PTSD and DSO domains: anxious arousal; depression; anger/irritability; intrusive experiences; defensive avoidance; dissociation; and impaired self-referencing.

## Discussion

### Summary of findings

**Figure 2:**
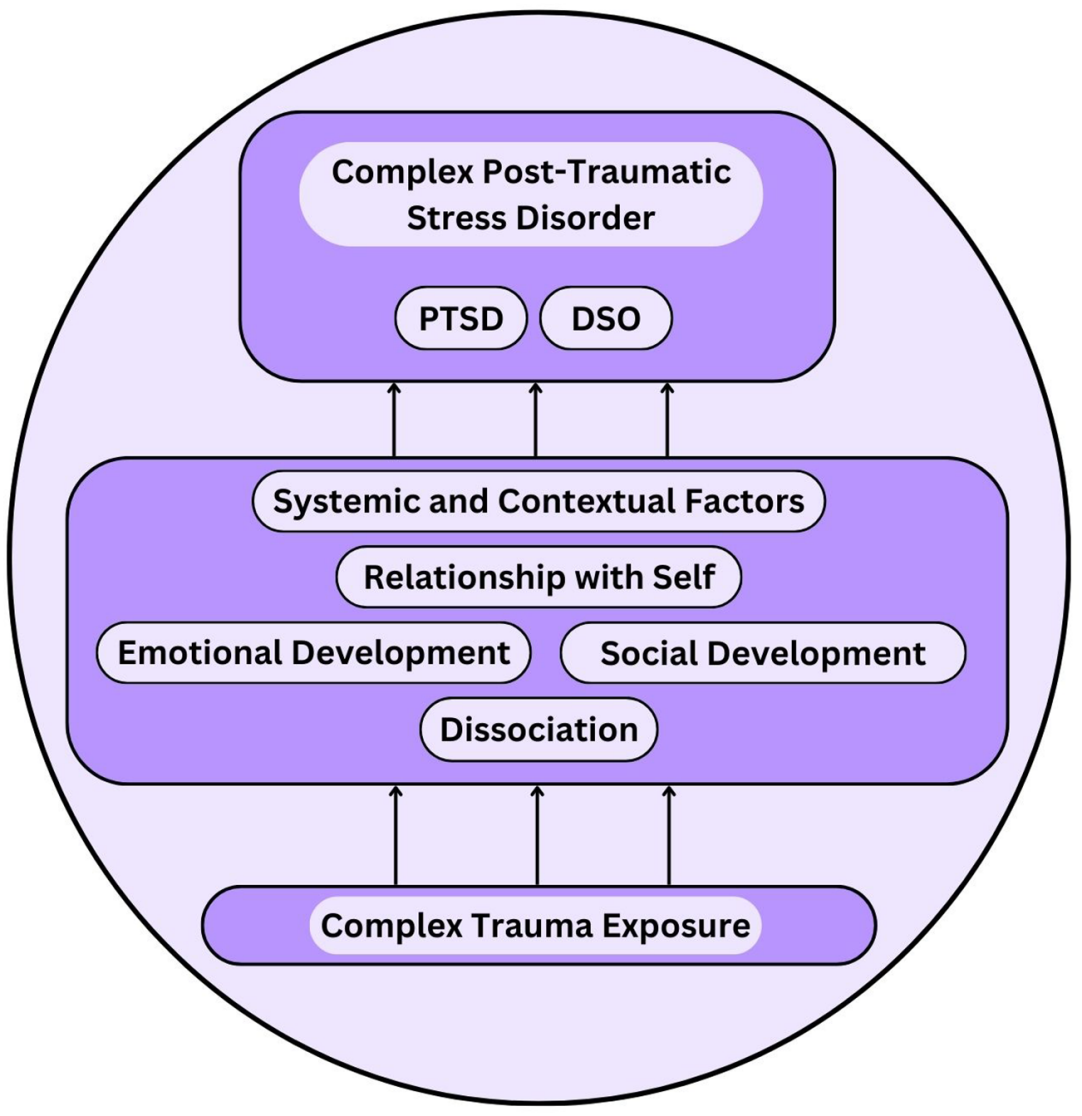
Conceptual multiple mediation model of the relationship between complex trauma exposure and complex post-traumatic stress disorder (CPTSD). Definitions: ‘PTSD’, Post-Traumatic Stress Disorder; ‘DSO’, Disturbances in Self-Organisation.

This is the first systematic review identifying factors which mediate the relationship between complex trauma in childhood and complex post-traumatic stress disorder (CPTSD). The findings indicate that a multitude of processes mediate this relationship: 1) dissociative processes, 2) an individual’s relationship to self, 3) emotional developmental processes, 4) social developmental processes, and 5) systemic factors contextualising the traumatised individual’s experience. These mediation effects were identified in clinical, at-risk and community samples across a variety of geographical locations. The mediating factors identified in this review are represented in a conceptual multiple mediation model in **Figure 2**.

### Comparison to previous research

The mediators identified in this review are supported by an extant literature examining the role of these processes in relation to both complex trauma exposure and CPTSD. Previously, dissociation has been conceptualised as a defensive biological capacity which acts as an ‘escape where there is no escape’ (75). It describes the process by which traumatic experiences are split off from consciousness and represented by dis-integrated fragments across different levels of the memory system (46), and is proposed to be responsible for the re-experiencing of trauma through ‘flashbacks’ (34; 76, 77). Additionally, in the context of complex trauma which frequently occurs within attachment relationships, it is likely that traumatic experiences in childhood are internalised as negative meanings about the self (78). Indeed, it has been proposed that childhood complex trauma should be viewed as a developmental process that results in a distorted self-concept (79). Furthermore, previous research suggests that such complex trauma occurring within attachment relationships would interrupt emotional development and the development of social cognition and social information processing (80–83). Lastly, systemic factors contextualising the experience of complex trauma have previously been found to play an important role in the development of CPTSD (20). This is not least because, by their nature, many forms of complex trauma (e.g. childhood abuse) occur within the contexts of relationships themselves.

To an extent, some of these mediating processes overlap with mediating processes involved in other clinical presentations, such as PTSD (i.e. dissociation, emotion dysregulation; 84; 85) and borderline personality disorder (‘BPD’; i.e. attachment insecurity; 86). It is possible that such processes reflect transdiagnostic mechanisms across these clinical presentations (87). Indeed, as PTSD is a required feature of the broader CPTSD construct, some overlap in mediating processes is to be expected; a meta-analysis has indicated the potential relevance of PTSD interventions in the treatment of CPTSD (16). Despite this, there are also differences in the mediating processes involved in CPTSD, PTSD and BPD. For example, this review identified one article which found that disconnection and impaired autonomy schemas acted as mediators in the association of complex trauma and CPTSD, whereas similar research examining BPD identified schemas of vulnerability to harm and defectiveness as mediators involved in the development of BPD (88). Additionally, another article in this review demonstrated that dissociative, emotional developmental and social developmental processed mediated complex trauma and CPTSD independently of BPD (43), thus indicating separate mediating pathways for CPTSD and BPD. This fits with previous research which has differentiated CPTSD and BPD as distinct constructs (25, 26). Further research will be required to determine which combinations of overlapping mediating processes interact to differentiate the development of each clinical presentation as either CPTSD, PTSD, or BPD.

More broadly, the findings of this review complement previous systematic reviews centred on CPTSD (11, 15, 16) by taking steps towards better understanding mediators of the relationship between complex trauma and CPTSD.

### Limitations of articles

All but two studies in this review were assessed as having moderate or weak methodological quality, largely employing cross-sectional designs which prevent casual inferences (89). This contributes to bias across studies; without longitudinal or experimental evidence, it is difficult to draw firm conclusions about the exact roles of each mediator in the pathways linking complex trauma and CPTSD. Furthermore, the lack of temporal precedence accounted for by cross-sectional designs can lead to difficulty in disambiguating the temporality of the mediation relationship (90). Despite this, atemporal statistical mediation effects were nevertheless demonstrated, thus indicating how the identified mediating factors explained the variance in CPTSD outcomes when accounting for the shared relationship between complex trauma, CPTSD and each mediating factor (90). In order to address this limitation, longitudinal research must be conducted in order to examine the replicability of the current findings within a temporal design, and to better understand the temporality of the established atemporal mediation relationships (90). This is particularly important when considering the conceptual overlap between several identified mediators (e.g. ‘Relationship to Self’) and CPTSD outcome domains (e.g. ‘Negative Self-Concept’), which poses difficulties in differentiating the identified mediating processes from CPTSD outcome domains.

One possible approach to understanding this at a conceptual level is through considering the difference between mediating processes and CPTSD outcome domains. For example, the ‘Relationship to Self’ category of mediators reflects a variety of maladaptive underlying processes (e.g. alterations in self-perception, self-judgement, early maladaptive schema) and protective processes (e.g. self-compassion, self-kindness) that were operationalised differently to how CPTSD outcomes were operationalised (i.e. through CPTSD-specific assessment measures) and interact to culminate in the outcome (i.e. a negative self-percept). This fits with previous research indicating the relevance of the identified mediating processes in the development of CPTSD (21; 18; 20; 17; 4, 19). As many studies utilised formal mediation analyses, this indicates that a mediation effect of these mediating variables influenced outcome variables at a statistical level (22). Despite this, as the studies included in this review operationalised mediator and outcome variables in cross-sectional study designs, it is difficult to disambiguate mediator and outcome variables beyond a conceptual level (91).

In order to more confidently conclude that the identified mediator variables are indeed mediators, as opposed to outcome variables, further research utilising longitudinal designs which can assess the temporality of relationships between variables will help to ensure the mediator and outcome variables are sufficiently disambiguated (92). Future research should involve examining the role of mediating factors in the relationship between complex trauma and CPTSD over at least two timepoints in order to establish the temporality and mechanistic nature of these mediation relationships.

Additionally, these studies relied on retrospective self-reports of complex trauma exposure; although the validity of these accounts is not in question, it is possible that the extent of trauma is under-reported (93). Furthermore, there was a lack of consideration given to the duration of complex trauma experiences. Despite this, some studies did account for the potential impact of confounding factors (e.g. gender, age) and showed that mediation effects were maintained in models which incorporated confounding factors. Longitudinal research is required to better understand the specific ways in which the mediators identified by studies in this review interact with complex trauma exposure in the development of CPTSD over time.

Additionally, although systemic factors relating to disclosure and acknowledgement of trauma within an individual’s system were identified, no studies examined the potential mediating role of wider systemic factors (e.g. community factors, poverty, discrimination). Furthermore, although studies were conducted across a wide range of cultural and geographical settings, only two studies collected data on the racial backgrounds of participants and five studies collected data on geographical background. No studies collected data on participant sexuality. It will be important for researchers to pay closer attention to variables such as race and sexuality due to minority stress and how experiences of minoritisation may moderate the relationship between complex trauma and CPTSD (94, 95). Examination of potential neurobiological and genetic mediators will also be of importance.

Lastly, the mediators identified through this review were tested across a range of studies. Future research should aim to assess the significance of these mediators in a single study, in order to examine the relative effects of each mediator along with potential interaction and cumulative effects. As the identified mediators are relevant to a range of clinical presentations, including PTSD and BPD, future research should also aim to identify which patterns of mediators may contribute to a particular outcome over another.

### Clinical implications

The identification of these mediators helps in better understanding possible underlying pathways and mechanisms involved in the development and prevention of CPTSD. Currently, in the United Kingdom, there is no ‘gold standard’ treatment recommendation for CPTSD (14). Although dissociation, emotion dysregulation, interpersonal difficulties and negative self-perception in CPTSD are noted as ‘barriers to engaging with trauma-focused therapies’ by NICE (14), these are not in and of themselves identified by NICE as targets for preventative action or therapeutic intervention for the alleviation of CPTSD itself. The findings of this review indicate that, beyond acting as barriers to engaging with trauma-focused therapies, it is possible these aspects of CPTSD could play an important mechanistic role in linking complex trauma and CPTSD and may be important targets for clinical intervention. However, further clinical research is required to examine whether targeting the mediators identified in this review could act as a mechanism for change and healing from complex trauma.

## Conclusions

There are many factors which mediate the relationship between complex trauma exposure in childhood and CPTSD. These mediators can be organised as processes relating to: 1) dissociation, 2) a disturbed relationship to self, 3) emotional development, 4) social development, and 5) systemic and contextual factors. Despite this, the methodological limitations of the studies which identified these mediating processes lead to difficulty in understanding the extent to which awareness of these mediating factors should inform prevention strategies, clinical formulation and intervention for CPTSD. This is particularly true when considering that these factors are not necessarily specific to CPTSD. Future longitudinal research is required to gain a deeper understanding of the possible developmental role of each mediating factor in the aetiology of CPTSD, and in examining the clinical utility of incorporating these mediators as targets for intervention in the treatment of CPTSD.

## Data availability statement

The original contributions presented in the study are included in the article/supplementary material. Further inquiries can be directed to the corresponding author.

## Author contributions

JH: Conceptualization, Data curation, Formal analysis, Methodology, Writing – original draft, Writing – review & editing. EL: Supervision, Writing – review & editing. VS: Supervision, Writing – review & editing.

## Appendix

**Appendix A:** Custom data extraction form used to extract data from each article.

	Participants	Design	Exposure Variable(s)	Outcome Variable(s)	Mediator Variable(s)	
First Author, Year, Country of Publication	**Population** **Sample** N (% Female) Mean Age (SD) Key Demographics	**Study Design** **Analysis**	**Complex Trauma Variables:** Trauma Type **Measure Type:** Measure	**CPTSD Outcomes**: % of Sample with CPTSD **Measure Type:** Measure	**Mediator Category:** Identified Mediator(s) **Measure Type:** Measure	**Results of Mediation Analyses** **Effect Size**
